# Development of a Humanistic Interprofessional Healthcare Model for a Regional Swiss Hospital Using Community-Based Participatory Action Research

**DOI:** 10.3390/ijerph23081047

**Published:** 2026-08-12

**Authors:** Catherine Bolduc, Virginie Renggli, Chrystel Carrupt, Filipa Pereira

**Affiliations:** 1 Valais Romand Hospital Centre, 1951 Sion, Switzerland; catherine.bolduc@hopitalvs.ch (C.B.); virginie.renggli@hopitalvs.ch (V.R.); chrystel.carrupt@gmail.com (C.C.); 2 School of Health Sciences, HES-SO Valais/Wallis, University of Applied Sciences and Arts Western Switzerland, 1950 Sion, Switzerland

**Keywords:** humanistic care, interprofessional collaboration, community-based participatory action research, patient experience, professional well-being, hospital model

## Abstract

**Highlights:**

**Public health relevance—How does this work relate to a public health issue?**

- The study addresses systemic pressures in hospital settings, including workforce shortages, burnout, and fragmented care, by proposing a humanistic interprofessional model grounded in frontline realities.
- It responds to public health challenges related to patient experience, care quality, and professional well-being within publicly funded healthcare systems.

**Public health significance—Why is this work of significance to public health?**

- This work demonstrates how a community-based participatory action research approach can generate an organisational care model aligned with public health values, such as equity, sustainability, and patient-centredness.
- It provides an empirically grounded framework linking humanistic care, interprofessional collaboration, and organisational culture in a real-world hospital network.

**Public health implications—What are the key implications or messages for practitioners, policy makers and/or researchers in public health?**

- For practitioners and managers, the model offers concrete, co-constructed levers to strengthen interprofessional collaboration while supporting professional well-being and care quality.
- For policy makers and researchers, it highlights the value of bottom-up, participatory approaches to designing sustainable hospital care models that integrate relational, organisational, and structural dimensions of health care.

**Abstract:**

Given the growing pressures on Switzerland’s healthcare system, developing innovative approaches that put humanism at the heart of care practices seems essential to respond to patients’ complex needs and to support healthcare professionals’ well-being. Although several frameworks address humanistic care or interprofessional collaboration, few have been co-developed with healthcare professionals to guide the implementation of both dimensions at the organisational level. This article explores the development of an interprofessional healthcare model aimed at integrating humanistic values into a public hospital network in a French-speaking region of Switzerland. This community-based participatory action research involved healthcare professionals representing different hospital departments and a patient feedback unit. The study focused on the co-development of an organisational model through three iterative phases: initiation (identification of needs), diagnosis (analysis of current practices) and design (development of strategies and solutions). The model was based on the three overarching themes: Care for Others and Oneself, Constructive Interactions, and Professional Excellence. The resulting model provides an organisational framework for integrating humanistic values into interprofessional practice in a hospital setting. Its implementation and evaluation will be required to assess its effects on patient experience, professional well-being, and organisational practices.

## 1. Introduction

Although Switzerland has one of the world’s most efficient healthcare systems, it faces major challenges, including economic and organisational constraints, demographic changes, staff shortages and increasing workloads [1]. These pressures contribute to fatigue and burnout among healthcare professionals [2,3], while also compromising the quality of patient-centred care, including the consideration of patients’ preferences and needs [3,4,5].

In this context, many healthcare professionals report a loss of meaning in their daily practice, often linked to a drift away from shared humanistic values [6,7]. This dissonance fosters feelings of distress, dissatisfaction and burnout, particularly when healthcare professionals perceive a misalignment between their care-related values and institutional priorities [8,9]. One of the primary causes of this discomfort lies in the tension between the desire to provide high-quality, personalised care and the strong institutional pressures for profitability and efficiency. When time and resources are constrained, opportunities for teamwork, shared decision making and coordinated, holistic patient care are reduced, further challenging healthcare professionals’ ability to align their practice with their humanistic values [6,7]. Healthcare organisations must therefore rethink their practices to better balance efficiency with collaborative, humanistic interprofessional care [1]. Strengthening interprofessional collaboration and supportive work environments is essential to improving both patient experience and healthcare professionals’ well-being while ensuring high-quality, patient-centred care [6]. In response to these challenges, healthcare organisations must rethink their practices to better balance efficiency with collaborative, humanistic interprofessional care [9,10,11].

Healthcare systems have evolved from paternalistic, disease-centred models to more collaborative, personalised approaches that prioritise patients’ holistic well-being [12]. Patient-centred care means providing humanistic care that is respectful of and responsive to individual patients’ preferences, needs, and values, and ensuring that patients’ values guide every clinical decision [13]. Humanistic care emphasises the ability to listen to patients’ needs and desires, understand their emotions, communicate with them and sense their values in life in order to develop therapeutic relationships [14,15]. When care becomes routine and devoid of personalisation, the quality of the interactions between carers and patients suffers, leading to a dehumanisation and fragmentation of the care process [9,16].

Interprofessional collaboration is considered a key determinant of high-quality, humanistic, patient-centred care [10,11]. The World Health Organization defines interprofessional collaboration as a practice where “multiple health workers from different professional backgrounds work together with patients, families, carers, and communities to deliver the highest quality of care across settings”, and it is recognised for its potential to improve patient outcomes, optimise resource use and enhance healthcare professionals’ job satisfaction [17]. Humanistic interprofessional care also enhances their motivation and well-being, maintaining their sense of professionalism and helping to prevent burnout [18]. However, achieving effective humanistic interprofessional collaboration in complex hospital environments remains challenging, as fragmentation, professional silos and institutional barriers often hinder the seamless coordination of care [19].

Many healthcare institutions have implemented competency-based frameworks to guide collaborative practice, with an emphasis on developing individual health professionals’ knowledge, skills and attributes [19]. While these frameworks have contributed to standardising collaborative skills, they primarily focus on individual competencies rather than on collective, team-based approaches. Evidence suggests that optimal interprofessional collaboration is best achieved through shared responsibilities and collective decision making within healthcare teams [20]. As such, shifting to team-based competency models is increasingly recognised as being essential to enhancing care delivery in hospital settings [21]. However, these frameworks provide limited guidance on how humanistic values can be embedded into organisational culture and co-developed with frontline healthcare professionals to support interprofessional practice in everyday hospital settings.

Responding to these challenges will require innovative, inclusive models that prioritise bottom-up approaches and enable frontline healthcare professionals to actively shape their shared vision of healthcare. To our knowledge, no organisational models have been developed through participatory approaches that explicitly integrate humanistic care and interprofessional collaboration at the hospital level [19,21]. To address this gap, the present study describes the bottom-up co-development of an organisational model designed to embed humanistic values into interprofessional hospital practice. Rather than proposing an additional conceptual or competency-based framework, the model translates humanistic and interprofessional principles into context-adapted organisational strategies co-developed with frontline healthcare professionals to support their integration into routine hospital practice.

## 2. Conceptual and Methodological Framework

Humanistic care has been conceptualised as a multidimensional construct encompassing relational, organisational and structural dimensions. Busch et al. [22], in their systematic review, identified these three complementary domains as essential for supporting humanistic healthcare. The relational domain emphasises empathy, respect for dignity, patient autonomy and recognition of the individual beyond the disease [16]. The organisational domain highlights the importance of supportive working conditions, recognition and leadership to enable healthcare professionals to provide ethical and compassionate care. Finally, the structural domain acknowledges the influence of the physical environment and organisational infrastructure on patient and staff experiences.

Unlike competency-based frameworks primarily focused on individual professionals’ knowledge and skills, the proposed model conceptualises humanistic interprofessional care as an organisational responsibility involving interactions, work environments and institutional conditions. These three domains provided the conceptual foundation for the participatory co-development of the proposed hospital model.

The model development process was guided by the Agency for Clinical Innovation that outlines the essential phases for developing and implementing innovative, evidence-based models of care. These phases include project initiation, diagnosis, solution design, implementation and sustainability [23]. The initiation phase involves identifying a need, building a rationale to justify change, and mobilising the necessary resources and support to advance in subsequent phases. The diagnosis phase focuses on defining the issue by analysing and understanding its root causes to address the actual problem rather than merely its symptoms. The solution design phase entails developing and selecting interventions to address the issues identified in the previous phase. The implementation phase is dedicated to translating necessary changes into practice to operationalise the hospital model developed. The sustainability phase aims to optimise the model’s long-term use by monitoring outcomes, assessing the impact of its implementation and making necessary adjustments. Each phase is essential to ensuring that the proposed care model meets patients’ health needs, remains economically viable and can be integrated into the existing healthcare system. The present article outlines the model’s development process from the initiation phase to the solution design phase.

## 3. Materials and Methods

### 3.1. Design

To develop the hospital model, we used a Community-Based Participatory Action Research (CBPAR) approach. This methodology actively involves all the stakeholders in every phase of the process, from problem identification to the development of solutions and the dissemination of results [23,24]. CBPAR ensures that every phase of the project is shared with its participants and validated by those directly involved in care delivery, enabling patients and healthcare professionals to play active roles in co-creating solutions. This methodology enhances project relevance and effectiveness by ensuring that solutions emerge directly from stakeholders’ needs and suggestions [24]. CBPAR was particularly appropriate because the objective was to co-develop an organisational model grounded in the experiences, priorities and contextual realities of the professionals and patients involved in hospital care.

The project team consisted of two Care Practice Development Managers employed by the CHVR and one Associate Professor from the School of Health Sciences, HES-SO Valais/Wallis. Throughout the project, the researchers adopted a facilitative rather than directive role, guiding discussions, encouraging equitable participation, and supporting the collaborative process while ensuring that priorities and solutions emerged from participants. Regular reflexive discussions within the project team encouraged critical reflection on emerging interpretations and helped minimise the influence of individual researchers’ perspectives on data collection, analysis and model development.

### 3.2. Study Setting

The Centre Hospitalier du Valais Romand (CHVR) is a public hospital network serving the French-speaking region of Valais across five sites: Sierre Hospital, Martigny Hospital, St-Amé Clinic, Malévoz Psychiatric Hospital and Sion Hospital. With a workforce of 3972 employees, the CHVR delivers care to 29,300 hospitalised patients and manages 502,714 outpatient consultations each year. The CHVR is part of the Valais Hospital, the entity that oversees the canton’s entire hospital system (in both French- and German-speaking regions).

### 3.3. Participants and Procedures

The healthcare professionals participating in the development of the hospital model were divided into distinct reflective, operationalisation and strategic groups, which were established to represent different perspectives and hierarchical levels within the CHVR and to ensure a representation of medical, nursing and allied health professionals (Table 1). Patients were represented by CHVR’s patient feedback unit “Espace patient”, a small in-house hospital team responsible for the collection and analysis of both anonymous and non-anonymous feedback from the CHVR’s patients and their relatives. Since a patient–partner programme was still under development at the CHVR, direct patient participation was not feasible at the time of the study. The CHVR’s patient feedback unit was therefore considered the most appropriate mechanism for integrating patients’ experiences and priorities into the model development process, while acknowledging that this represented an indirect rather than direct form of patient participation. The Reflective Group (RG), formed using purposive sampling, consisted of 42 healthcare professionals providing direct patient care. They were identified by their department heads to create an interprofessional group representative of the entire CHVR. Each department head proposed three to five healthcare professionals, ensuring diverse representation across professions and hospital sites. Four physicians were also invited to join the group. Participation was voluntary, and invited professionals were free to accept or decline the invitation. This approach fostered a heterogeneous group composition, encompassing all the CHVR sites and a broad spectrum of healthcare professions.

The Operationalisation Group (OG) comprised 15 proximity managers (also selected by their department heads) and a senior physician. The Strategic Group (SG) consisted of ward heads and clinical nurse specialists.

### 3.4. Data Collection and Project Development

Within the CBPAR design framework and given the large number of participants, a combination of data collection methods was used to ensure methodological rigour while promoting active stakeholder participation. First, collaborative meetings were used to gather diverse perspectives and co-develop tailored solutions. These meetings were facilitated to encourage equitable participation and open discussion among participants. Activities were primarily conducted in small interprofessional groups, and the separation of the Reflective, Operationalisation, and Strategic Groups according to their organisational roles helped minimise direct hierarchical influence during discussions. Second, participants produced written outputs, including concept maps, flipcharts and worksheets, which were collected at the end of each workshop. Concept maps facilitated a visual representation of ideas and the connections between emerging themes. Third, the three project team members systematically recorded field notes to document contextual observations and key discussion points throughout the workshops. Participants’ written outputs and project team field notes constituted the raw data for the thematic analysis and were compiled into textual documents for in-depth analysis. Audio recordings were not used, as the project focused on the collective production of ideas rather than individual narratives and involved a large number of participants. All workshops followed predefined facilitation guides prepared by the project team to ensure consistency in data collection across participant groups. The combination of collaborative meetings, concept maps and project team field notes enabled data triangulation, thereby strengthening the validity of our findings while adhering to the core principles of CBPAR.

Thus, to adhere to the CBPAR approach and ensure that the data collected during the diagnostic and solution design phases had genuinely emerged from the participants, the project team implemented a number of workshops guided by the Agency for Clinical Innovation framework [23].

The diagnostic phase, conducted in November 2023 with the RG and CHVR’s patient feedback unit, aimed to analyse current care practices and highlight interprofessional collaboration and patient-centred care. The first 60-min workshop focused on sharing patients’ testimonials, followed by a debriefing session designed to highlight the challenges and opportunities of interprofessional collaboration and patient-centred care. A second 120-min workshop brought together participants in groups of five to identify concrete aspects of their current practices that could be improved, guided by the main questions of ‘What do we need?’ and ‘What can we do?’.

Between November 2023 and January 2024, the project team performed a thematic analysis of the collected data. This phase enabled the identification of themes and subthemes emerging from the collected data.

The solution design phase was based on the diagnostic phase and involved multiple steps. Each step built on the outputs of the previous one, creating an iterative cycle of reflection, feedback, refinement and co-development throughout the model development process. In January 2024, two workshops were conducted with the RG. Workshop 3 (90 min) focused on member confirmability to validate the themes and subthemes identified in the thematic analysis and to ensure the robustness and relevance of the findings by integrating participants’ perspectives. Participants were actively involved in this process, initially working in small groups to add, modify, or remove themes and subthemes, before coming together as a large group to reach a collective validation. Workshop 4, held on the same day, brought together all 42 members of the RG, randomly divided into three subgroups, to develop action strategies aligned with the results of the diagnostic phase. In turn, each subgroup worked on all of the three focus areas previously identified, proposing actions. A member of the project team facilitated each discussion. These 120-min workshops resulted in concrete, actionable interventions for implementing solutions in clinical practice.

The solution design phase continued into March 2024 with Workshop 5, where the RG developed strategies to enhance humanistic interprofessional healthcare all along patients’ hospital care pathways. Working in groups of ten, participants analysed a fictional clinical vignette developed for this study and inspired by common clinical situations (Supplementary File S1) that provided a concrete framework for discussion and guided the development of practical interventions at different stages along the patient’s care pathway. This approach promoted meaningful, collective, patient-centred reflections that actively engaged diverse healthcare professionals in the co-design of solutions.

The process continued in May 2024 by involving the OG in two complementary workshops to translate the proposed strategies into concrete, priority actions. Workshop 6 focused on prioritising subthemes and action strategies using the Eisenhower matrix, a tool that classified the RG’s proposals according to their level of impact and importance [25]. However, this workshop did not work as intended, as everything was considered a priority. Workshop 7 used a participatory World Café approach to foster dynamic discussions within small groups of 4 or 5 participants [26]. This facilitated the development of concrete, tangible interventions to operationalise the priority subthemes and action strategies. The discussions encouraged creativity and participant engagement while ensuring alignment with the overall strategic objectives defined.

The implementation and sustainability phases are currently in development to facilitate the project’s optimal dissemination and long-term integration across the hospital’s various sectors and professional groups. However, these two phases fall outside the scope of this manuscript. Table 2 provides a chronological overview of the project development and data collection process across the first three phases of the model.

### 3.5. Data Analysis

Abductive thematic analysis was used to analyse the collected data [27]. This method was particularly suited to this project as it enables the development of a hospital model that is both theoretically informed and empirically validated [28]. Positioned between the inductive and deductive approaches, the abductive method integrates empirical data and existing theoretical frameworks. Unlike induction, which generates theories from data, or deduction, which tests predefined hypotheses, abduction seeks plausible explanations for observed phenomena by reconciling empirical findings with established theories. This approach fosters a dynamic interaction between empirical and theoretical data [29]. Consequently, our hospital model was refined based on feedback from various stakeholders yet remained grounded in the principles of patient-centred interprofessional collaborative practices.

The abductive approach used in this project went through several key phases [28]. Data collected from collaborative meetings, concept mapping and the project team’s field notes were transcribed for in-depth analysis. This first step enabled the team to familiarise themselves with the data and identify areas requiring further exploration. Next, the data were manually independently coded by two researchers to identify meaningful units and emerging themes [27]. Coding decisions were compared during regular project meetings, and differences in interpretation were resolved through discussion until consensus was reached. The third project team member contributed to these discussions whenever necessary. Codes with similar meanings were iteratively grouped into subthemes, which were subsequently organised into broader themes and overarching themes by comparing patterns across the different data sources. Although some concepts (e.g., communication, collaboration and professional roles) appeared across several themes, thematic boundaries were determined according to the primary meaning attributed to each concept within its context. These boundaries were refined through repeated comparison of codes, patterns and their contextual meaning through iterative discussions within the project team and subsequent workshops in which participants actively reviewed, modified, merged, added or removed themes and subthemes, thereby contributing directly to the refinement of the conceptual framework.

Throughout the analysis, abductive reasoning was used to iteratively compare the emerging findings with existing theory. The themes were subsequently interpreted through the lens of humanistic patient-centred interprofessional care [14,15,22], allowing the conceptual model to be co-refined by researchers and participants while preserving the uniqueness of the empirical data.

The four criteria of scientific rigour, as defined by Lincoln and Guba [30], were also applied throughout the project (Table 3). Credibility was strengthened through data and investigator triangulation, iterative discussions within the project team, and continued data collection until no substantially new themes emerged. Data saturation was considered to have been achieved when successive workshops no longer generated substantially new themes or actions, and the emerging conceptual model remained stable despite further discussions. Confirmability was ensured by validating emerging themes and the evolving model using participant feedback from the RG in Workshops 2 and 3 [31]. This approach ensured that the themes identified and the model proposed accurately reflected participants’ experiences and perspectives, thereby strengthening the credibility and reliability of the analysis. Moreover, it fostered active collaboration between the project team and participants, enabling the co-construction of knowledge and refinement of the model based on participants’ feedback.

### 3.6. Ethical Considerations

Given the CBPAR design of this study, in which no interventions were performed nor sensitive personal health data collected, formal ethics committee approval was not required under the Swiss Federal Act on Research Involving Human Beings (LRH, 810.30, Art. 2, al. 2b–c). The study was conducted in accordance with the Declaration of Helsinki, and all fundamental ethical principles—including respect for participants’ autonomy, confidentiality, transparency, and informed consent—were strictly upheld. Participants were provided with information regarding the objectives, methods, and implications of the study and gave their oral consent to participate. Written consent was not required due to the minimal risk and the collaborative nature of the CBPAR framework, which actively engages voluntary participants in co-creating solutions [32].

## 4. Results

The collected data of proposed actions by the RG and OG were condensed into subthemes, which were then grouped into seven themes categorised under the three main overarching themes of Care for Others and Oneself, Constructive Interactions and Professional Excellence. Table 4 summarises the results obtained.

The three overarching themes reflected participants’ shared experiences and priorities regarding humanistic interprofessional care. Care for Others and Oneself emphasised the need to preserve the human dimensions of care despite increasing organisational pressures. During Workshop 1, one participant stated, “Healthcare professionals are human too; we must not forget their human side,” while another questioned, “How can we restore meaning? Does it make sense to restore meaning without changing the environment?”- reflecting participants’ concerns that preserving the human dimensions of care requires a supportive organisational context. Constructive Interactions highlighted the central role of communication and collaboration in ensuring high-quality care. Participants in Workshop 2 highlighted that “A care intervention that is not communicated is like a care intervention that never happened” and that “Communication needs to be learned and practised at every level.”. Finally, Professional Excellence emerged as a shared aspiration among participants to combine clinical expertise with humanistic values. As expressed by one participant group during Workshop 4: “Working together with kindness and respect to provide effective, high-quality care.”.

**Figure 1:**
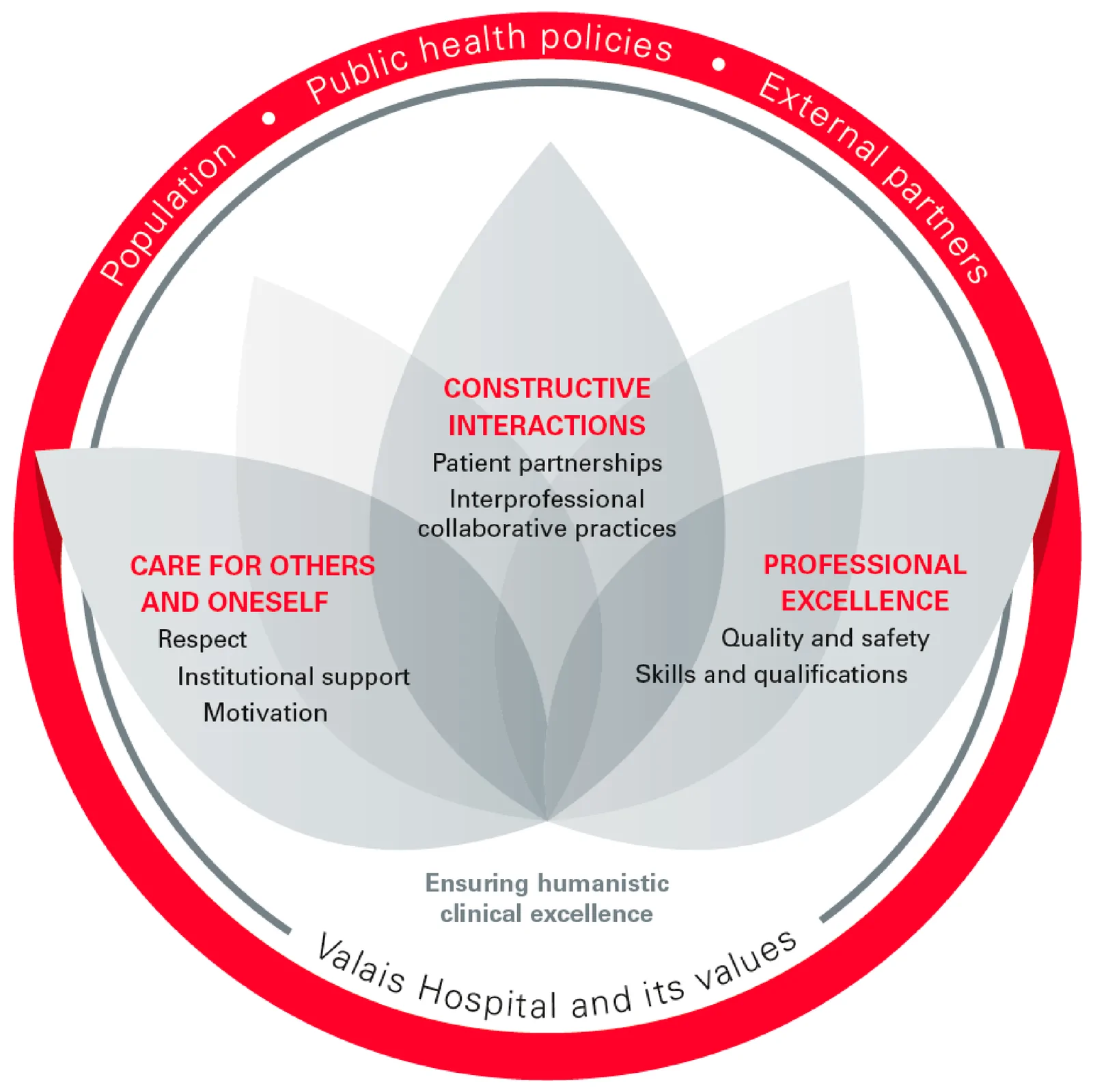
Diagram representing the CHVR Hospital Care Model developed using a CBPAR approach.

The model developed was named the CHVR Hospital Care Model and is illustrated in Figure 1 with its three main overarching themes and seven themes derived from our CBPAR.

The overlapping petals illustrate how the three overarching themes interweave and interact with each other to create an active, dynamic, interprofessional work culture. The overlap highlights how actions in one area positively influence those in others.

The model is based on a shared foundation composed of the core values of the Valais Hospital: care relationship, responsibility, equity, a spirit of collaboration, and sustainability. These values guide all actions, behaviours, and resources mobilised to enable all hospital stakeholders, whether caregivers or non-caregivers, to contribute effectively to patient care.

The three overarching themes are framed by a red circle, symbolising the influence of external factors specific to the context and environment of this hospital network’s healthcare practices: the local population whose needs must be addressed by integrating its social and cultural specificities; the public health policies at the local and national levels that shape the hospital’s practices and priorities; and its external partners, such as outside institutions and care networks, that help ensure the continuity and quality of services. This model is grounded in the values of the Valais Hospital, with the CHVR being an integral part of its framework.

A slogan was developed for the model, informed by the results: Ensuring humanistic clinical excellence. The CHVR Hospital Care Model reflects the participants’ commitment to combining clinical expertise with humanistic values by encouraging collaboration between all of the hospital’s stakeholders in the quest for excellence that is not limited solely to technical skills but also embraces the relational and human dimensions of care.

The action strategies proposed by the Reflective and Operationalisation Groups to operationalise the subthemes identified through the thematic analysis are presented in Supplementary File S2.

## 5. Discussion

This article outlines the first three phases in the development of a hospital care model based on interprofessional collaborative practices and humanistic care, using a CBPAR approach [33]. Following these phases facilitated the integration of the perspectives of hospital staff and patients while taking into account the contextual dynamics and specific needs of the various clinical settings within the hospital. The originality of the CHVR Hospital Care Model lies not in redefining the principles of humanistic or interprofessional care, but in translating these principles into concrete, context-specific organisational actions that reflect the realities and priorities of the local hospital environment. Unlike existing conceptual or competency-based frameworks [19,21,22], the CHVR Hospital Care Model focuses on organisational transformation rather than individual competencies and integrates humanistic care and interprofessional collaboration within a single co-developed framework. Through the CBPAR process, these principles were translated into context-adapted organisational actions that can support their implementation in routine hospital practice. The approach adopted promoted an iterative, inclusive co-construction process that centred on collaboration between the project team and stakeholders, thus ensuring that the model was tailored to the context’s practical realities and aligned with the different stakeholders’ shared values.

The iterative nature of the CBPAR process was particularly evident during the prioritisation stage. Participants initially viewed nearly all emerging actions as equally important, illustrating the complexity of translating a broad range of perspectives into a coherent organisational model. Subsequent discussions enabled the refinement and prioritisation of these actions.

The results highlighted three main overarching themes that reflect the relational, organisational and structural domains of humanistic care identified by Busch et al. [22]. Our findings suggest that these domains are closely interconnected and should be integrated, rather than considered as separate dimensions, to foster humanistic interprofessional care in hospital settings. Extending Busch et al.’s framework, the CHVR Hospital Care Model operationalises these domains into three complementary overarching themes—Care for Others and Oneself, Constructive Interactions, and Professional Excellence—that provide an organisational framework for embedding humanistic interprofessional care into everyday practice.

Our findings also highlight the inherent tension between organisational efficiency and humanistic care. Instead of positioning these as opposing objectives, the co-developed model suggests that strengthening collaboration, supportive organisational practices and shared values may help reconcile operational performance with humanistic practice in everyday hospital care.

### 5.1. Care for Others and Oneself

The overarching theme of Care for Others and Oneself addresses the importance of recognising and fulfilling individuals’ emotional, physical and psychological needs, whether they are patients or professionals. Several authors have emphasised that the care provided to patients should not overshadow professionals’ self-care, which is essential to maintaining their ability to provide care without putting themselves at risk [34]. And yet, self-care is often undervalued despite its crucial role in preserving healthcare professionals’ well-being and the quality of hospital care [34].

Three themes were grouped under this overarching theme. The participants perceived Respect to be a particular act of care aimed at valuing colleagues’ qualities, skills and positive attributes while acknowledging their specific contributions. These results were in line with existing knowledge, which describes a respectful environment as one where colleagues become aware of each other’s strengths and the challenges they face, while reinforcing individuals’ performance through positive feedback [35].

In our results, the theme of Institutional support refers to the policies, resources and structures that are deployed by an institution to provide systematic support to its staff. This confirmed existing knowledge, emphasising the importance of this support in guaranteeing an environment favourable to effective care and team resilience [36]. However, our data also provided additional insights into participants’ specific expectations regarding this support, particularly in terms of occupational medicine, valuing professionals’ lived experiences, improving workstation ergonomics or integrating resources such as volunteers.

Motivation was defined as a process that activates, guides and maintains individuals’ behaviours with regard to achieving expected objectives. The participants particularly emphasised the importance of promoting pleasure at work and strengthening team dynamics to maintain a positive climate. This vision of motivation must be part of an overall organisational approach, as Albenhasnan’s [37] systematic review showed by highlighting that employee motivation was not the result of a simple cause-and-effect relationship but rather the result of a complex interaction of elements.

### 5.2. Constructive Interactions

Constructive Interactions were described as respectful, collaborative, caring interactions between different stakeholders: patients, relatives and healthcare professionals. Such interactions were perceived to improve both the quality of care and patient safety, while also contributing to professionals’ well-being by giving their work a sense of purpose.

These findings aligned with the work by McLaney et al. [21], who identified effective communication, coordination, mutual respect and shared empathy as the pillars of successful interactions. Furthermore, Busch et al. [22] and McLaney et al. [21] highlighted the crucial roles of teamwork, collaboration and constructive conflict management in ensuring effective interactions within hospitals. Our results enriched this knowledge by revealing the concrete practices and relational mechanisms specific to this context, thus making it possible to translate these principles into actions that have a direct impact on stakeholders’ satisfaction and well-being (Supplementary File S2).

The two main themes of Patient partnerships and Interprofessional collaborative practices fall under this overarching theme. According to the study’s participants, the former is characterised by encouraging patients to play an active role in decision-making and in their own care. Patients and informal caregivers are encouraged to share their preferences, values, knowledge and concerns with healthcare professionals, thereby promoting a collaborative approach. These findings were in line with existing knowledge that highlights this partnership’s importance in improving the quality of care and patients’ experiences [38]. This collaborative process, where professionals are responsible for providing transparent, accessible information, enables patients to make informed decisions and feel more involved all along their care pathway.

The second theme of Interprofessional collaborative practices was described as an active process of collaboration in which healthcare professionals share not only their skills but also their experiences and perspectives. These interactions make it possible to coordinate care better and respond to patients’ needs more effectively. Scientific evidence shows that when interprofessional care teams work more collaboratively, they can improve the delivery of patient-centred care, leading to better outcomes for patients and health systems [39]. This study highlighted the challenges teams face when implementing such collaboration, including managing professionals’ differences of opinion or approach.

### 5.3. Professional Excellence

The third overarching theme identified was Professional Excellence, highlighting the importance of expertise based on mastery of the knowledge and skills acquired during one’s initial training and perfected during one’s professional development. Participants saw this expertise as essential for solving complex problems and providing quality patient care. Indeed, according to the literature, professional expertise makes it possible to make informed, evidence-based decisions that are adapted to the continuous evolution of knowledge in the healthcare field [40]. Professional excellence requires employees to engage in critical thinking and continuous learning in order to constantly readjust their practices to the latest evidence, thereby guaranteeing the quality of care and patient safety [41].

This overarching theme encompasses the two related themes of Quality and safety and Skills and qualifications. Quality of care is defined as a hospital’s ability to increase the likelihood of achieving its patients’ desired states of health, whereas patient safety focuses on the absence of avoidable harm and the minimisation of any unnecessary risks associated with that care [42]. Our results complemented these definitions by highlighting the specific, tangible dimensions that our participants perceived to be essential, such as avoiding interruptions and optimising hospital logistics.

Skills and qualifications were identified as a central theme, highlighting the importance of the overall composition and distribution of skills and qualifications within interprofessional teams. The literature defines this theme as the need to balance available skills with the demands of care to ensure effective and quality care [43]. The present study’s results reflected its participants’ specific expectations. They underlined the need to recognise and exploit each professional’s specific skills so as to enhance collective effectiveness. They also highlighted the importance of clarifying each staff member’s role in order to reduce ambiguity, improve interprofessional collaboration and optimise the coordination of care.

Based on these findings, proposals were formulated to foster a more humanistic interprofessional culture across our hospital network. The participants believed it was essential to develop an institutional approach that valued humanism and was shared by all those involved in delivering care. This culture should encourage mutual trust with a view to fostering an environment where both carers and patients feel respected and heard. Although humanism is a complex concept, it is important to define its associated behaviours in everyday practice. Integrating these behaviours into an institutional culture can help to create a sustainable humanistic environment. Our results also highlighted the importance of how an interprofessional philosophy can encourage collaboration between disciplines, enriching exchanges and ensuring more personalised care, tailored to the specific needs of each patient and their relatives. The participants placed a particular emphasis on the need to support their commitment, ensure that their working conditions were conducive to their personal development and recognise their essential contributions to the quality of care. It is essential, therefore, that hospitals consider the needs of their professional staff in terms of their training, emotional support and well-being at work. It is important to set aside time and put in place interventions that encourage this development, whether it is via training, reflection workshops or discussion forums. These will enable hospital healthcare professionals to adapt to patients’ changing needs while remaining rooted in humanistic approaches to delivering care.

The participatory CBPAR approach was instrumental in shaping this model. By engaging healthcare professionals, managers, and patient representatives in an iterative process of reflection, validation, and co-design, the model evolved to reflect the realities, priorities, and organisational context of the CHVR. This collaborative approach enhanced the relevance, acceptability, and feasibility of the proposed actions. Nevertheless, the co-construction process remained primarily driven by healthcare professionals, with patient perspectives being represented indirectly through the hospital’s patient feedback unit. Future implementation and refinement of the model should therefore involve patient partners directly to further strengthen its participatory and patient-centred dimensions. Moreover, translating the model into routine practice may face important organisational challenges, including limited time for interprofessional exchanges, competing institutional priorities, resource constraints, staff turnover, and resistance to organisational change. Addressing these challenges will require sustained leadership, organisational commitment, and continued stakeholder engagement. A key implementation challenge will be integrating the model into routine hospital practice while reconciling operational demands with the time and resources needed to sustain humanistic interprofessional collaboration. Beyond its initial implementation, ensuring long-term organisational change will also require regular evaluation and adaptation so that the model remains aligned with evolving organisational needs and priorities. Future studies should focus on assessing how effective the deployment of the CHVR Hospital Care Model was in developing humanistic care practices in terms of patients’ experiences, professional well-being and interprofessional collaboration.

### 5.4. Limitations

Although this study made significant contributions to our knowledge, it had certain limitations. Patients were represented by CHVR’s patient feedback unit, an in-house hospital team dedicated to collecting feedback from hospitalised patients. Although the research team unanimously considered this to be the most efficient way of gathering patients’ experiences and preferences during the model’s development, the absence of direct patient involvement represents an important limitation. Direct participation by patients would likely have enriched the co-construction process by providing first-hand insights into their experiences, expectations and priorities regarding humanistic care. Consequently, some patient priorities may have been interpreted through the institutional perspective of the patient feedback unit rather than emerging directly from patients’ own discussions. Future phases of the project should actively involve patient partners to strengthen the participatory nature of the model and ensure that patients contribute directly to its refinement, implementation and evaluation.

Regarding the participation of healthcare professionals, our purposive sampling method may have introduced a selection bias. Although participation was voluntary, healthcare professionals were initially identified by their department heads, which may have favoured individuals who were more engaged with organisational initiatives or more motivated to participate in the project. Consequently, the perspectives collected may not fully reflect those of the broader hospital workforce. Moreover, despite the measures taken to promote open and equitable participation throughout the workshops, the influence of organisational culture and existing professional norms on participants’ contributions cannot be completely excluded. In addition, for feasibility reasons, not all CHVR staff were fully represented. The process was conducted exclusively with healthcare professionals, even though the model also includes non-professional support teams. Involving non-clinical support staff would be essential to fully capture the model’s scope and to ensure its comprehensive implementation. A future quantitative approach would be necessary to document staff perceptions in a more exhaustive and representative way.

Our model was developed using a CBPAR approach specifically adapted to the organisational culture and context of the CHVR hospital network. Consequently, its transferability to other contexts or healthcare establishments remains uncertain and should be considered with caution, taking into account differences in organisational culture, governance and local contextual factors. Adaptation through a new participatory process would likely be required before implementation in other settings.

Finally, the present article focuses solely on the first three phases of the CHVR Hospital Care Model’s development. The subsequent phases, particularly those of implementing and sustaining the model, are also essential to the model’s overall effectiveness and will be crucial to assessing its impact on hospital practice. These phases will also enable the evaluation of the organisational mechanisms through which the model influences practice, as well as its effects on patient experience, healthcare professionals’ well-being, and other organisational outcomes.

## 6. Conclusions

The present study used a bottom-up approach to develop a humanistic interprofessional healthcare model for a regional public hospital network, taking full account of the contemporary challenges facing Switzerland’s healthcare system. Framed by three overarching themes and seven themes, the model provides a conceptual framework to support the integration of humanistic values into interprofessional hospital practice. It offers a shared language and aligned objectives that may facilitate interprofessional collaboration and coordination in complex healthcare settings. Our findings highlight the importance of integrating relational, organisational and structural dimensions when developing humanistic hospital practices. More broadly, this study demonstrates the value of participatory co-construction for developing context-adapted organisational models that reflect stakeholders’ experiences, priorities and organisational realities. This article reports the first three phases of the model’s development and provides a foundation for its future implementation and evaluation. Further research is needed to assess implementation processes and the model’s impact on patient experience, healthcare professionals’ well-being, interprofessional collaboration, and organisational outcomes.

## Figures and Tables

**Table 1:** Project participants’ roles and responsibilities.

Roles	Composition	Responsibilities
Project mandataries	Director of Nursing Medical Director Director of the CHVR	Needs identification Project representation Decision making Resource allocation
Project Team 3 members	2 Care Practice Development Managers 1 Associate Professor at the School of Health Sciences, HES-SO Valais/Wallis	Defining the problem (identifying root causes) Supporting solutions development Overall coordination Task planning and monitoring overall progress Communication (informing and engaging with stakeholders) Report writing Ensuring quality and consistency Supporting the model’s implementation
Reflective Group (RG) 42 members	25 Nurses 4 Seniors Physicians 3 Healthcare Assistants 2 Radiology Technicians 2 Physiotherapists 1 Nutritionist	Assessing institutional needs and the current context Defining interprofessional patient-centred values and needs Translating these values into daily clinical practices Developing solutions
CHVR’s patient feedback unit	Patients and relatives represented by CHVR’s patient feedback unit	Contributing to the proposed model by sharing patients’ and/or relatives’ experiences Promoting interprofessional patient-centred care all along patients’ hospital care pathways
Operationalisation Group (OG) 16 members	15 Proximity Managers 1 Senior Physician	Defining the model’s priority actions Integrating the model’s actions into daily clinical practices Aligning values with the institution’s overall objectives and daily management challenges
Strategic Group (SG) 17 members	8 Head department nurses of different clinical specialities across all of the CHVR’s sites 3 Hospital Site Directors 1 Human Resources Director 5 Clinical Nurse Specialists 1 Senior Resident Physician 1 Head Physician	Strategic planning and actions for model implementation Integrating the new model into the institutional framework to ensure its sustainability and long-term success Embedding the new model into daily clinical practices

**Table 2:** Chronological overview of the project development and data collection process.

Project Phase [23]	Participants	Timeline	Activities	Description of Activities
Initiation phase	Project Team	June 2023 to November 2023	Work sessions	Creating the project team and clarifying needs and the context.
Diagnostic phase	Reflective Group and CHVR’s patient feedback unit	10 November 2023	Workshop 1	Sharing two patients’ testimonials on negative hospital care experiences, followed by a debriefing (60 min).
			Workshop 2	Group work with five participants to identify concrete aspects of their current practices that need improvement by answering the questions ‘What do I need?’ and ‘What could we do?’, followed by a group discussion (total of 120 min).
	Project Team	November 2023 to January 2024	Work sessions	Thematic data analysis.
Solution design phase	Reflective Group	19 January 2024	Workshop 3	The RG validated the member confirmability of themes and subthemes derived from the thematic data analysis (90 min).
			Workshop 4	Co-developing action strategies based on the validated subthemes and themes identified during the diagnostic phase. The workshop was structured around the validated themes emerging from the diagnostic phase, facilitated by the project team and included the RG’s 42 members, who were randomly assigned to the three different groups (total of 120 min). Each group contributed in turn to the different thematic areas by proposing action strategies.
	Reflective Group	22 March 2024	Workshop 5	Based on clinical vignettes, working groups of ten participants developed strategies for making care pathways more interprofessional and patient-centred throughout hospital stays (Supplementary File S1) (120 min).
	Operationalisation Group	24 May 2024	Workshop 6	Prioritising subthemes and action strategies using the Eisenhower matrix [25] to consider their levels of impact and importance (60 min).
			Workshop 7	Using the World Café method [26] to propose interventions to operationalise the priority subthemes and action strategies, conducted in groups of four or five participants (120 min).
	Strategic Group	6 June 2024	Debate	Presenting the project’s progress and approving the model developed (90 min).

**Table 3:** Scientific rigour criteria applied in the project [27,30].

Criteria of Scientific Rigour	Strategies Applied
Credibility	Heterogeneously composed participant groups (RG, OG and SG); Data collected from the RG, OG and SG through collaborative meetings, concept mapping and project team field notes; Continuing data collection and iterative analysis until no substantially new themes emerged (data saturation); Conducting pre- and post-workshop debriefings by the project team; Investigator triangulation through independent coding and regular consensus discussions; Externally verifying credibility outside the project team and RG to ensure results and interpretations were well-founded.
Dependability	Holding regular weekly coordination meetings (triangulation and process tracking); Coding data precisely; Keeping detailed field notes.
Confirmability	Member checking through validation of the emerging themes and evolving model (between workshops); Evaluating the data collection and analysis process by the project team members.
Transferability	Using purposeful sampling with inclusion and exclusion criteria to ensure maximal professional and demographic representativeness; Describing guidelines and collected data comprehensively and in detail.

**Table 4:** Subthemes, themes and overarching themes that emerged from the analysis of the collected data.

Overarching theme: Care for Others and Oneself			
Respect	Institutional Support		Motivation
Adopting efficient and caring communication Expressing limits Fostering a sense of trust Ensuring transparency	Having a dedicated occupational health service Dealing with work-related accidents and providing the necessary support Encouraging healthcare professionals to express their experiences Ensuring a better understanding of mental burdens at work Integrating resources: volunteers, carers, ethical advice, etc. Improving ergonomics for carers		Enhancing enjoyment at work and strengthening team dynamics Encouraging personal commitment Encouraging benevolent leadership Implementing an enhanced onboarding programme for new employees
Overarching theme: Constructive Interactions			
Patient partnerships		Interprofessional collaborative practices	
Improving patients’ welcome at admission Encouraging patients to make their own decisions Developing personalised care plans Involving relatives and informal caregivers Ensuring safety during discharges, departures or transfers		Understanding each other’s roles Ensuring good collaboration within inter-professional teams through mutual help and support Setting up briefings and debriefings Optimising communication Fostering collaboration across units and services	
Overarching theme: Professional Excellence			
Quality and safety		Skills and qualifications	
Avoiding interruptions Optimising hospital logistics Providing care with rigour and efficiency		Recognising and using each professional’s specific skills Clarifying roles	

## Data Availability

The data presented in this study will be made available by the corresponding author upon reasonable request for research purposes, subject to institutional approvals.

## Supplementary Materials

The following supporting information can be downloaded at: https://www.mdpi.com/article/10.3390/ijerph23081047/s1, Supplementary File S1: Clinical Vignette; Supplementary File S2: CHVR Hospital Care Model actions proposed by the Reflective and the Operationalisation Groups.

## Abbreviations

The following abbreviations are used in this manuscript:

CBPAR	Community-Based Participatory Action Research
CHVR	Centre Hospitalier du Valais Romand
OG	Operationalisation Group
RG	Reflective Group
SG	Strategic Group
