# Supplementary material for: Development of a Humanistic Interprofessional Healthcare Model for a Regional Swiss Hospital Using Community-Based Participatory Action Research
Source: Int J Environ Res Public Health. 2026 Aug 12;23(8):1047. doi: 10.3390/ijerph23081047 (PMC13512711; doi:10.3390/ijerph23081047)
Supplement: Supplementary file 1 [file ijerph-23-01047-s001.zip › Supplementary File S1.pdf]

Supplementary File S1: Clinical vignette

Note: This clinical vignette is fictional, was developed specifically for this study, and does not represent an actual patient.

21:00
Mr. Junod, 82 years old, arrives on foot at the Emergency Department in Sion, accompanied by his daughter. She cannot stay with him because she has young children at home and her husband is away for work. At triage, Mr. Junod explains to the nurse that he came to consult for worsening shortness of breath this evening, with a persistent cough for the past 7 days and new greenish sputum since this morning. The nurse asks him administrative questions, takes his vital signs, and performs a brief clinical assessment in order to evaluate the urgency and severity of his dyspnea. She then asks him to wait in the waiting room.

23:00
Mr. Junod has been in the waiting room for an hour with other patients. He goes to the nursing desk and asks if he will be seen by a doctor soon, as he has now been waiting for 2 hours. A passing nurse replies that he will be seen as soon as possible but that they have many patients and all stretchers are occupied. Mr. Junod sighs, returns to his seat, and waits.

00:00
A nurse invites him into a cubicle and asks him to settle in so that the doctor can come and evaluate him. Mr. Junod feels cold but does not say anything, afraid of disturbing.

00:15
The physician arrives; she has a soft and calm voice. She takes his history, asking questions about his symptoms and health background. She then performs a physical examination. She informs him that the nurse will come to collect a sputum culture, do a blood test, and that he will need a chest X-ray. Mr. Junod feels reassured even though he is not entirely sure what to expect. At least things are moving forward, he thinks.

00:30
The nurse arrives and chats with him about the bad weather and the heavy workload these days. She has accepted to cover for a sick colleague tonight. Since she received the call at 4 pm, she has not slept today. She is beginning to feel the fatigue, but remains alert and notices that the patient has a slight neck retraction, probably due to his seated position with his head slightly tilted back. She helps him sit more comfortably. She rechecks his oxygen saturation: 88%. She puts him on oxygen without explaining why. Mr. Junod finds her kind and trusts her. He does not ask any questions. He appreciates her presence and the informal conversation that reassures him.

00:50
The orderly, who knows the emergency service well and feels useful helping his colleagues, enters his cubicle and asks: "You're doing well, Mr. Junod?" He nods, and without further explanation, the orderly begins pushing the stretcher. He likes being recognized for his efficiency. In the X-ray room, the radiology technician approaches Mr. Junod, introduces herself, checks his ID bracelet, and asks him to stand up. She explains— for the tenth time that evening— that he must press his chest against the plate with arms raised. She positions him correctly, goes into her booth, and instructs him to inhale deeply and hold his breath for a few seconds. She then returns, tells him the exam is over, and invites him back onto his bed. The orderly brings him back to his cubicle. Mr. Junod then falls asleep, sitting on his stretcher.

03:00
After discussing the case with the nurse at the desk, the physician goes to Mr. Junod's room. She wakes him to tell him that he will be admitted to the medical ward for pneumonia. She briefly explains that he will need to start intravenous antibiotics and that the duration of hospitalization will depend on his condition. Before leaving, she asks if he has any questions. He replies that it is not necessary to be hospitalized for "just a pneumonia." He tries to convince her by saying that his wife is at home and could take care of him. The physician, slightly irritated at having to justify her decision, explains that his oxygen saturation is borderline, that he needs oxygen, and that the infection is clearly present in his lungs. To help him recover, he must receive IV medication. The nurse then arrives to insert a peripheral IV line and start the antibiotic treatment. She briefly asks him about his living situation, family, and usual state of health, before leaving the room to complete the documentation.

06:50
The bed management team informs the emergency staff that a spot has opened up in the medical ward and that Mr. Junod must be transferred quickly to relieve overcrowding in the ED. A nurse assistant quickly gathers his personal belongings.

07:00
The orderly arrives. Mr. Junod is transferred upstairs. A brief handover is done between the night-shift ED nurse and the day-shift medical ward nurse.

07:10
The nurse welcoming Mr. Junod introduces herself as Sarah. She seems preoccupied and not very available. She has just finished a very busy night shift. She is exhausted. She quickly settles him in his room before returning to finish her handover. Mr. Junod senses that she has no time, so he does not dare ask for help to arrange his belongings or to locate the toilets. He feels weak, as he has slept little and is sick. He is also worried because his wife is home alone and he has not given her any news—nor his daughter. He had not wanted to wake them during the night, but now he imagines they must be worried. He gets up barefoot to reach his luggage and look for his phone. He falls. His roommate calls for help, and a staff member comes to assist him. "Sir, you must not get up alone. Next time, please ask for help," he tells him. This staff member, whose name Mr. Junod cannot read clearly on the badge, asks him several questions and performs a physical assessment. Fortunately, Mr. Junod is not injured. The physician ensures that the call bell is within reach and says he will return later. The doctor would like to give feedback on the admission to the nurse in charge, but the nurse replies that he is already very behind schedule and suggests discussing it during ward rounds unless there is something urgent. In his room, a bit shaken, Mr. Junod takes a moment to regain his composure. It has been a lot for him.

07:40
Breakfast is served. He asks the young woman delivering the tray if she can help him find his phone. She asks where he left it. Mr. Junod replies he doesn't know, that it must be in his luggage. She says she needs to finish serving trays and will come back later to help. She has a heavy workload: the ward is full, she is the only nurse assistant today, she must also supervise a student, and handle logistics. Mr. Junod waits until after breakfast, but the young woman does not return. It is the student who comes to collect his tray. Mr. Junod does not dare ask for help again.

08:10
A nurse brings him his medication. He asks about his social support, his apartment, medical history, allergies, lifestyle habits, and assesses his respiratory condition. Mr. Junod takes the opportunity to again express that he disagrees with this hospitalization and does not understand why it is necessary. He asks whether someone has called his wife. The nurse replies that it is his responsibility to do so. In

a dry tone, Mr. Junod insists that someone must explain to his wife why he is hospitalized, as he himself does not know. The nurse repeats that he needs oxygen and intravenous antibiotics. Mr. Junod asks if he may use their phone. The nurse remains calm, informing him he will return with the service phone before leaving the room.

08:40
The nurse, feeling satisfied, comes back with the service phone. He is proud that he thought of picking it up on his way, despite his very long to-do list at this hour. He hands the phone to Mr. Junod and leaves quickly to continue his work. To his dismay, Mr. Junod cannot remember his wife's or daughter's number. He begins to feel short of breath again. His breathing becomes faster, and he starts to feel dizzy. He rings the call bell.

A nurse who has just completed training in clinical assessment responds to the call and notes Mr. Junod's respiratory distress. She is pleased to put her new knowledge into practice. She begins appropriate care: takes his vital signs, asks about his symptoms, examines his chest, and listens to lung sounds with her stethoscope. She leaves the room and gives a SCAR-structured handover to the nurse in charge, who returns with the equipment to administer a nebulizer treatment. Mr. Junod sits semi-upright with a Venturi mask on his face. A tear falls as he finds himself alone. Finally, his breathing calms and returns to normal. He feels better again and falls asleep.

10:00
Instruction: What could happen next?
At 10:00, you must go to Mr. Junod for a physiotherapy session (airway clearance) and to assess his walking ability. What will you be attentive to?

For the synthesis: Identify 5 simple ways to make the care relationship throughout Mr. Junod's hospital journey more humanizing.
