# Supplementary material for: Development of a Humanistic Interprofessional Healthcare Model for a Regional Swiss Hospital Using Community-Based Participatory Action Research
Source: Int J Environ Res Public Health. 2026 Aug 12;23(8):1047. doi: 10.3390/ijerph23081047 (PMC13512711; doi:10.3390/ijerph23081047)
Supplement: Supplementary file 1 [file ijerph-23-01047-s001.zip › Supplementary File S2.pdf]

Supplementary File S2: CHVR Hospital Care Model actions proposed by the Reflective and the Operationalisation Groups

Overarching theme: Care for Others and Oneself	
Theme : Respect	
1.1	Promote positive, non-violent communication
1.2	Support expression of lived experience and provide spaces for listening
1.3	Ensure that managers are available for their staff
1.4	Establish a culture of constructive feedback
1.5	Foster teamwork
1.6	Develop a service charter reflecting the team's values
1.7	Organize training sessions on intergenerational understanding
1.8	Organize training in leadership, mediation, and conflict management
1.9	Adopt a zero-tolerance policy for harassment and violence
1.10	Ensure equity in roles and responsibilities
Theme : Institutional Support	
2.1	Support skills development for roles with responsibilities
2.2	Improve workstation ergonomics
2.3	Encourage collaboration between caregivers and support departments (HR, IT, training)
2.4	Adopt a task prioritization tool and create protected time by revising priorities
2.5	Implement an objective workload management system, including a daily emotional check-in (e.g. red/green indicators)
2.6	Offer health check-ups and fast-track access to occupational health services in case of work-related issues
2.7	Provide legal support and employer facilitation in case of workplace accidents
2.8	Free up time for peer discussion groups, debriefings, and clinical situation reviews, with safe spaces both within and outside the unit
2.9	Offer a daycare center adapted to all employee schedules
2.10	Publish an internal newsletter (Gazette News CHVR) to inform and connect staff
2.11	Support skills development for roles with responsibilities
Theme : Motivation	
3.1	Encourage recognition of achievements and career development projects
3.2	Strengthen the onboarding program for new staff
3.3	Set up a suggestion box to collect improvement ideas
3.4	Encourage feedback from new employees on their first impressions
3.5	Reinforce positivity by expressing appreciation toward colleagues
3.6	Create a relaxing environment with music
3.7	Highlight work through positive patient feedback
3.8	Develop peer/mentor roles to support new team members
3.9	Recognize staff involvement in internal projects or training
3.10	Provide more training opportunities (workshops, seminars, conferences) within the hospital
3.11	Involve team members in working groups
3.12	Set departmental objectives
3.13	Organize an annual motivational interview
3.14	Redefine the time allocated to management duties for each manager
3.15	Create an "open-door" space with the line manager

3.16	Organize monthly team outings with designated “friendly organizers” from the team
3.17	Acknowledge important moments in staff members’ lives (birth, birthdays, bereavement) and recognize their commitment

**Overarching theme:** ***Constructive Interactions***	
Theme : Patient partnerships	
4.1	Strengthen training involving patient partners (e.g. therapeutic patient education, motivational interviewing, breaking bad news)
4.2	Involve patients in CHVR projects (patient-partners)
4.3	Mobilize available resources: volunteers, care aides, associations, ethics committees, listening spaces, etc.
4.4	Implement standardized admission and discharge protocols with adapted checklists
4.5	Ensure bedside handovers and joint medical-caregiver rounds
4.6	Use a communication board in patient rooms and define care goals together with the patient
4.7	Identify and involve informal caregivers in patient care
4.8	Inform patients about the service's rules and functioning
4.9	Display a poster introducing the care team within the service
4.10	Consider patients’ needs and preferences by asking questions such as “What is important to you?” and respecting their individual wishes
4.11	Take the necessary time and ensure safety during patient admission and discharge
4.12	Anticipate patient transfers and movements
Theme : Interprofessional collaborative practices	
5.1	Organize shadowing sessions (“Walk in my shoes”) to foster cross-sector understanding
5.2	Establish a shared language using tools such as TeamSTEPPS, checklists, and interprofessional meetings
5.3	Schedule interprofessional exchanges (e.g. huddles) and weekly medical-caregiver meetings
5.4	Ensure verbal handovers among caregivers, both within and across sites
5.5	Plan interprofessional activities and team-building sessions
5.6	Assign appropriate roles to optimize time management
5.7	Use a communication board in patient rooms
5.8	Ensure familiarity with the service’s schedule (handover and visiting times)
5.9	Organize off-site interprofessional activities to strengthen team cohesion
5.10	Centralize and simplify access to patient information
5.11	Strengthen interdepartmental relationships to encourage collaboration
5.12	Implement a shared patient record accessible across intra- and extra-hospital interprofessional teams
5.13	Increase coordination meetings with the bed management team
5.14	Promote interprofessional training
5.15	Develop service-level projects through interprofessional collaboration
5.16	Ensure everyone understands their job descriptions and roles within the team
5.17	Introduce briefing and debriefing practices after problematic situations
5.18	Coordinate and support patients in complex situations
5.19	Encourage joint physician-nurse admissions for improved care delivery

Overarching theme: Professional Excellence
Theme : Quality and safety
6.1 Develop peer/mentor roles and value the expertise of experienced caregivers
6.2 Minimize interruptions during nursing handovers and ensure a calm environment for these exchanges
6.3 Optimize skill distribution with 12-hour work shifts
6.4 Encourage staff participation in working groups
6.5 Promote a culture of constructive feedback
6.6 Inform patients about service rules and functioning
6.7 Improve clinical information traceability
6.8 Reduce interruptions during care by informing patients about routines and using voicemail when staff are unavailable
6.9 Introduce the use of tablets to facilitate communication and data access
6.10 Provide interview rooms adapted to the needs of patients and staff
6.11 Simplify the hospital's digital systems for ease of use
6.12 Align medical and nursing practices
6.13 Regularly update coordinated protocols through interprofessional collaboration
6.14 Encourage staff participation in morbidity and mortality conferences
6.15 Promote a culture of error analysis to learn from critical situations
Theme : Skills and qualifications
7.1 Review job descriptions and competency frameworks
7.2 Highlight each person’s specific skills (e.g., registered nurses, medical assistants)
7.3 Identify and formalize a list of institutional competencies (e.g., reference persons for port-a-cath, hypnosis)
7.4 Develop mobile competency units (e.g., wound care nurses, palliative care specialists)
7.5 Standardize delegated tasks across the hospital
7.6 Offer continuing education in therapeutic education, motivational interviewing, and breaking bad news
7.7 Encourage participation in courses and conferences
7.8 Develop service-level projects aligned with hospital training offerings
7.9 Increase the number of internal patient transport staff
7.10 Implement neutral supervision systems
7.11 Update the list of staff who speak various languages in the Phoenix system
7.12 Assign roles optimally to improve time management
7.13 Share team member roles clearly within the teams
7.14 Implement service organization based on skills and workload distribution, aligned with field needs
7.15 Recognize prior learning and support career development projects
7.16 Encourage mutual support within and across departments

Note. Numbers are used only to help identify actions, and do not reflect an order of priority or importance. While some of the proposed actions reflect aspirations and creative thinking from participants, others are already implemented in certain settings and are no longer perceived as innovative. These actions should not be interpreted as prescriptive or evidence-based recommendations; rather, their implementation will be guided by local priorities and, where possible, supported by a review of the existing literature.
